# The clinical usefulness of extravascular lung water and pulmonary vascular permeability index to diagnose and characterize pulmonary edema: a prospective multicenter study on the quantitative differential diagnostic definition for acute lung injury/acute respiratory distress syndrome

**DOI:** 10.1186/cc11898

**Published:** 2012-12-11

**Authors:** Shigeki Kushimoto, Yasuhiko Taira, Yasuhide Kitazawa, Kazuo Okuchi, Teruo Sakamoto, Hiroyasu Ishikura, Tomoyuki Endo, Satoshi Yamanouchi, Takashi Tagami, Junko Yamaguchi, Kazuhide Yoshikawa, Manabu Sugita, Yoichi Kase, Takashi Kanemura, Hiroyuki Takahashi, Yuichi Kuroki, Hiroo Izumino, Hiroshi Rinka, Ryutarou Seo, Makoto Takatori, Tadashi Kaneko, Toshiaki Nakamura, Takayuki Irahara, Nobuyuki Saito, Akihiro Watanabe

**Affiliations:** 1 Division of Emergency Medicine, Tohoku University Graduate School of Medicine, 1-1 Seiryo-machi, Aoba-ku, Sendai 980-8574, Japan; 2 Department of Emergency and Critical Care Medicine, St. Marianna University School of Medicine, 2-16-1 Sugao, Miyamae, Kawasaki, Kanagawa 216-8511, Japan; 3 Department of Emergency and Critical Care Medicine, Kansai Medical University, 10-15 Fumizono-cho, Moriguchi City, Osaka 570-8506, Japan; 4 Department of Emergency and Critical Care Medicine, Nara Medical University, 840 Shinjo-cho, Kashihara, Nara 634-8521, Japan; 5 Department of Emergency and Critical Care Medicine, Kurume University School of Medicine, 67 Asahi-machi, Kurume-shi, Fukuoka 830-0011, Japan; 6 Department of Emergency and Critical Care Medicine, Faculty of Medicine, Fukuoka University, 7-45-1 Nanakuma, Jonan-ku, Fukuoka City, Fukuoka 814-0180, Japan; 7 Advanced Emergency and Critical Care Center, Tohoku University Hospital, 1-1 Seiryo-machi, Aoba-ku, Sendai 980-8574, Japan; 8 Department of Emergency and Critical Care Medicine, Nippon Medical School Hospital, 1-1-5 Sendagi, Bunkyo-ku, Tokyo 113-8603, Japan; 9 Department of Emergency and Critical Care Medicine, Aidu Chuo Hospital, 1-1 Tsuruga, Aiduwakamatsu, Fukushima 965-8611, Japan; 10 Division of Emergency and Critical Care Medicine, Department of Acute Medicine, Nihon University School of Medicine, 30-1 Oyaguchi kamimachi, Itabashi-ku, Tokyo 173-8610, Japan; 11 Shock Trauma and Emergency Medical Center, Tokyo Medical and Dental University Hospital, 1-5-45 Yushima, Bunkyo-ku, Tokyo 113-8519, Japan; 12 Department of Emergency and Critical Care Medicine, Juntendo University Nerima Hospital, 3-1-10 Takanodai, Nerima-ku, Tokyo 177-8521, Japan; 13 Department of Critical Care Medicine, Jikei University School of Medicine, 3-19-18 Nishi-shinbashi, Minato-ku, Tokyo 105-8471, Japan; 14 Emergency and Critical Care Medicine, National Hospital Organization Disaster Medical Center, 3256 Midori-cho, Tachikawa-shi, Tokyo 190-0014, Japan; 15 Department of Intensive Care Medicine, Saiseikai Yokohamashi Tobu Hospital, 3-6-1 Shimosumiyosi, Tsurumi-ku, Yokohama City, Kanagawa 230-8765, Japan; 16 Department of Emergency and Critical Care Medicine, Social Insurance Chukyo Hospital, 1-1-10 Sanjo, Minami-ku, Nagoya City, Aichi 457-8510, Japan; 17 Advanced Emergency and Critical Care Center, Kansai Medical University Takii Hospital, 10-15 Fumizono-machi, Moriguchi Cty, Osaka 570-8507, Japan; 18 Emergency and Critical Care Medical Center, Osaka City General Hospital, 2-13-22, Miyakojima Hondori, Miyakojima, Osaka 534-0021, Japan; 19 Department of Anesthesia, Kobe City Medical Center General Hospital, 2-2-1 Minatojimaminamimachi, Chuo-ku, Kobe City, Hyogo 650-0046, Japan; 20 Department of Anesthesia and Intensive Care, Hiroshima City Hospital, 7-33 Motomachi, Naka-ku, Hiroshima-shi, Hiroshima 730-8518, Japan; 21 Advanced Medical Emergency and Critical Care Center, Yamaguchi University Hospital, 1-1-1 Minamikogushi, Ube City, Yamaguchi 755-8505, Japan; 22 Intensive Care Unit, Nagasaki University Hospital, 1-7-1 Sakamoto, Nagasaki 852-8501, Japan; 23 Department of Emergency and Critical Care Medicine, Nippon Medical School Tama Nagayama Hospital, 1-7-1 Nagayama, Tama-shi, Tokyo 206-8512, Japan; 24 Department of Emergency and Critical Care Medicine, Nippon Medical School Chiba Hokusoh Hospital, 1715 Kamagari, Inzai-shi, Chiba 270-1694, Japan

## Abstract

**Introduction:**

Acute lung injury (ALI)/acute respiratory distress syndrome (ARDS) is characterized by features other than increased pulmonary vascular permeability. Pulmonary vascular permeability combined with increased extravascular lung water content has been considered a quantitative diagnostic criterion of ALI/ARDS. This prospective, multi-institutional, observational study aimed to clarify the clinical pathophysiological features of ALI/ARDS and establish its quantitative diagnostic criteria.

**Methods:**

The extravascular lung water index (EVLWI) and the pulmonary vascular permeability index (PVPI) were measured using the transpulmonary thermodilution method in 266 patients with PaO_2_/FiO_2 _ratio ≤ 300 mmHg and bilateral infiltration on chest radiography, in 23 ICUs of academic tertiary referral hospitals. Pulmonary edema was defined as EVLWI ≥ 10 ml/kg. Three experts retrospectively determined the pathophysiological features of respiratory insufficiency by considering the patients' history, clinical presentation, chest computed tomography and radiography, echocardiography, EVLWI and brain natriuretic peptide level, and the time course of all preceding findings under systemic and respiratory therapy.

**Results:**

Patients were divided into the following three categories on the basis of the pathophysiological diagnostic differentiation of respiratory insufficiency: ALI/ARDS, cardiogenic edema, and pleural effusion with atelectasis, which were noted in 207 patients, 26 patients, and 33 patients, respectively. EVLWI was greater in ALI/ARDS and cardiogenic edema patients than in patients with pleural effusion with atelectasis (18.5 ± 6.8, 14.4 ± 4.0, and 8.3 ± 2.1, respectively; *P *< 0.01). PVPI was higher in ALI/ARDS patients than in cardiogenic edema or pleural effusion with atelectasis patients (3.2 ± 1.4, 2.0 ± 0.8, and 1.6 ± 0.5; *P *< 0.01). In ALI/ARDS patients, EVLWI increased with increasing pulmonary vascular permeability (*r *= 0.729, *P *< 0.01) and was weakly correlated with intrathoracic blood volume (*r *= 0.236, *P *< 0.01). EVLWI was weakly correlated with the PaO_2_/FiO_2 _ratio in the ALI/ARDS and cardiogenic edema patients. A PVPI value of 2.6 to 2.85 provided a definitive diagnosis of ALI/ARDS (specificity, 0.90 to 0.95), and a value < 1.7 ruled out an ALI/ARDS diagnosis (specificity, 0.95).

**Conclusion:**

PVPI may be a useful quantitative diagnostic tool for ARDS in patients with hypoxemic respiratory failure and radiographic infiltrates.

**Trial registration:**

UMIN-CTR ID UMIN000003627

## Introduction

Pulmonary edema is characterized by the abnormal accumulation of fluid in the extravascular space of the lungs and is a common finding in critically ill patients [1]. This pathological condition may develop due to an increase in the pulmonary capillary permeability (acute lung injury (ALI), acute respiratory distress syndrome (ARDS)), an increase in the pulmonary capillary hydrostatic pressure (hydrostatic or cardiogenic pulmonary edema), or both [2]. Pulmonary edema can be detected by clinical evaluation of factors such as patients' history, physical findings, and routine laboratory examinations, and is confirmed by the presence of bilateral pulmonary infiltration on chest radiography [2,3]. However, interpretation of these factors is often limited by a certain degree of subjectivity that might cause inter-observer variation even among experts, particularly in critically ill patients [4-6]. Moreover, intensive care physicians may find it difficult to determine the cause of the extravascular lung water (EVLW) increase [7].

In 1994 the American Thoracic Society and the European Society of Intensive Care Medicine co-published the proceedings of a consensus conference on ARDS, and defined ALI and ARDS as an American-European Consensus Conference (AECC) definition [8,9]. Although many clinical trials performed after the publication of the proceedings used the AECC definition, this definition has been suggested to have various issues, including a lack of explicit criteria for defining what is acute, the sensitivity of the PaO_2_/FiO_2 _(P/F) ratio to different ventilator settings, poor reliability of the chest radiograph criterion, and difficulties distinguishing hydrostatic edema [10-14]. These criteria are also not sensitive predictors of disease severity and outcomes [4,5,15-17] because: the P/F ratio varies considerably across different FiO_2 _levels, particularly when FiO_2 _< 0.5, PaO_2 _> 100 mmHg, or when the shunt fraction is low; many patients who initially fulfill the ARDS criteria might improve the P/F ratio > 200 mmHg after application of positive end-expiratory pressure for a short time or the use of higher FiO_2_; and hypoxemia in ARDS may also be related to atelectasis or a low cardiac output [14]. Based on these limitations, a novel definition has been proposed that takes into account the clinical and physiologic characteristics of ALI/ARDS [18]. The Berlin definition for ARDS was published recently and was demonstrated to have better predictive validity for mortality than the AECC definition [10]. Although ARDS has been described as a type of acute, diffuse inflammatory lung injury leading to increased pulmonary vascular permeability, increased lung weight, and loss of aerated lung tissue, as the panel agreed in their conceptual model, none of the suggested criteria evaluates the increase in pulmonary microvascular permeability, a hallmark of ARDS [10]. Not only the AECC definition but also the Berlin definition may include an extensive range of respiratory insufficiencies without an increase in pulmonary microvascular permeability.

Previous studies have reported on methods of quantifying pulmonary edema [19,20]. The double-indicator thermodilution technique allows the measurement of EVLW, and excellent correlation between *in vivo *and postmortem gravimetric EVLW values was obtained in both animal and human studies using this method [21,22]. However, this method is cumbersome and technically challenging for routine clinical application. The single-indicator technique is therefore used in clinical settings; this method is as sensitive as the double-indicator technique [23,24]. We previously validated the accuracy of EVLW measurements obtained using the single-indicator technique in the postmortem lung samples and defined the statistically normal EVLW values in a human autopsy study [25]. The close relationship between EVLW and outcome has been also demonstrated [26].

The transpulmonary thermodilution technique provides an estimation of both EVLW and the pulmonary blood volume, and the ratio of these two parameters - the pulmonary vascular permeability index (PVPI) - has been shown to reflect the pulmonary microvascular permeability [7,27].

Increased pulmonary vascular permeability is the crucial pathophysiological feature of ALI/ARDS and has been considered a quantitative diagnostic criterion for ALI/ARDS [28]. PVPI has been evaluated to enable one to differentiate ALI/ARDS from hydrostatic edema [7]. PVPI was shown to be useful for determining the mechanism of pulmonary edema in ALI/ARDS, and PVPI ≥ 3 allowed the diagnosis of ALI/ARDS with a sensitivity of 85% and a specificity of 100%. However, that study was a single-center retrospective review of only 48 patients (ALI/ARDS, 36 patients; hydrostatic edema, 12 patients).

The aims of this study were to clarify the clinical pathophysiological features of ALI/ARDS, and to establish the quantitative differential criteria of ALI/ARDS on the basis of PVPI assessed using the transpulmonary single thermodilution technique.

## Materials and methods

This prospective, observational, multi-institutional study was approved by the ethics committee of each of the 23 institutions, and written informed consent was provided by all patients' next of kin. The study was registered with the University Hospital Medical Information Network Clinical Trials Registry: UMIN-CTR ID UMIN000003627.

### Patients

Between March 2009 and August 2011, 301 patients from 23 critical care centers at tertiary care hospitals were enrolled in this study. In all of 23 participating institutions, the single transpulmonary thermodilution technique is one of the standard monitoring methods for circulatory and respiratory management of critically ill patients. The median (interquartile range) number of included patients per each institution was 10 (6 to 18).

The inclusion criteria were aged older than 15 years, requiring mechanical ventilation (expected over 48 hours) for acute respiratory failure with a P/F ratio ≤ 300 mmHg and bilateral infiltration on chest radiography and transpulmonary thermodilution technique monitoring of circulatory/respiratory status as per the attending physician's discretion. Exclusion criteria were as follows: over 5 days from the onset of acute respiratory failure with a P/F ratio ≤ 300; chronic respiratory insufficiency (chronic obstructive pulmonary disease, and so forth); history of pulmonary resection/pneumonectomy, pulmonary thromboembolism, and severe peripheral arterial disease; cardiogenic shock with a cardiac index < 1.5 l/minute/m^2^; acute phase of trauma with lung contusion and burns; other causes rendering patients unsuitable for evaluation with the transpulmonary thermodilution technique, including patients with moderate to severe valvopathy; and the attending physician identifying patients as inappropriate for inclusion.

**Figure 1:**
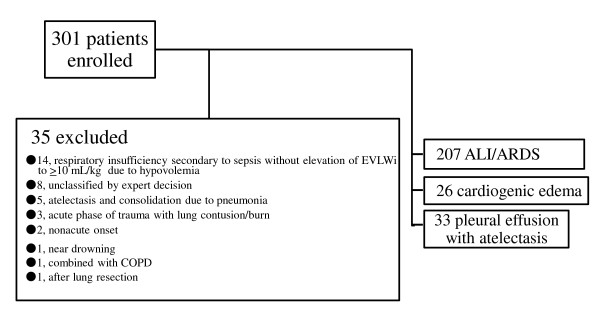
**Patient enrollment, exclusion, and classification**. ALI, acute lung injury; ARDS, acute respiratory distress syndrome; COPD, chronic obstructive pulmonary disorder; EVLWI, extravascular lung water index.

Of the 301 enrolled patients, 266 were included in this analysis. Reasons for the exclusion of 35 patients are shown in Figure 1.

In this study, the diagnosis of pulmonary edema was established on the basis of: the presence of bilateral infiltrates on chest radiography; P/F ratio ≤ 300 mmHg; and an increase in the EVLW indexed to the predicted body weight (extravascular lung water index (EVLWI)) ≥ 10 ml/kg. Although there is no definitive quantitative criterion of EVLWI for pulmonary edema, we recently reported that the normal EVLWI value is approximately 7.4 ± 3.3 ml/kg for humans [25]. EVLWI ≥ 10 ml/kg was used for definition of pulmonary edema in the previously reported study [1,29].

### Measurement of EVLWI and PVPI using the transpulmonary thermodilution method

A 4-Fr or 5-Fr femoral arterial or 4-Fr brachial arterial thermistor-tipped catheter (PV2014L16N, PV2015L20N, or PV2014L22N; Pulsion Medical Systems, Munich, Germany) was inserted in all patients by the attending physicians' discretion and connected to the PiCCO^® ^plus or PiCCO^® ^2 monitor (Pulsion Medical Systems). The monitor uses a single-thermal indicator technique to calculate the cardiac output (CO), global end-diastolic volume (GEDV), EVLW, and other volumetric parameters. A 15 ml bolus of 5% glucose at 5°C was injected through a central venous catheter, and the CO calculated using the Stewart-Hamilton method. A 15 ml bolus dose was selected as previously described [25] and the precision of measurements has been demonstrated [30,31]. The central venous catheters were accessed from the jugular or subclavian route in all patients. Concurrently, the mean transit time and exponential downslope time of the transpulmonary thermodilution curve were calculated. The product of CO and mean transit time represents the intrathoracic thermal volume [23]. The product of CO and exponential downslope time represents the pulmonary thermal volume [32]. GEDV is calculated as the difference between the intrathoracic thermal volume and the pulmonary thermal volume, and represents the combined end-diastolic volumes of the four cardiac chambers. The intrathoracic blood volume (ITBV) is calculated as the linear relationship with the GEDV [23]:

ITBV = 1.25 × GEDV - 28.4

EVLW is the difference between the intrathoracic thermal volume and ITBV [23,32]. The detailed principles and calculations involved in deriving EVLW using the thermodilution technique are discussed elsewhere [7,33]. PVPI is calculated as the ratio of EVLW and pulmonary blood volume [7]. ITBV and GEDV are indexed to the body surface area.

The median EVLW value was obtained after three bolus injections of 15 ml each [31]. The absolute EVLW value was indexed to predicted body weight, calculated as 50 + 0.91 (height (cm) - 152.4) for males and 45.5 + 0.91 (height (cm) - 152.5) for females [34]. For indexing EVLW, the predicted body weight instead of the actual body weight was used because: lung volumes are dependent on gender and height, not on weight [35]; measurement of EVLW indexed to the actual body weight can be underestimated in obese patients [36,37]; and the EVLWI has been shown to be a better prognostic indicator than EVLW indexed to the actual body weight [38-40]. The results were analyzed using PiCCO-VoLEF Data Acquisition for Win32 Version 6.0 for PiCCO^® ^plus or Version 2.0.0.13 for PiCCO^® ^2 (Pulsion Medical Systems).

### Assessment of circulatory/respiratory status, other parameters, and clinical course

At the time of enrollment (day 0), the clinical conditions, cause of respiratory insufficiency, Acute Physiology and Chronic Health Evaluation II score, Sequential Organ Failure Assessment score, and Lung Injury Scale score were assessed [41,42]. Echocardiography was performed to measure the left ventricular ejection fraction, left ventricular end-diastolic dimension, interventricular septum thickness, E/A ratio, left atrial dimension, inferior vena cava diameter and its respiratory variation, presence of hypo/akinesis, valvular abnormality, left ventricular systolic/diastolic function, and the thermodilution hemodynamic assessment validity. Chest computed tomography was also conducted on the day of enrollment. B-type natriuretic peptide (BNP) or *N*-terminal pro-BNP was measured on the day of enrollment and daily thereafter.

From the day of enrollment to day 2, the circulatory/respiratory status and other parameters except for routine clinical workup were assessed; the clinical course, including respirator settings, Lung Injury Scale score [43], Sequential Organ Failure Assessment score, antithrombin activity level, serum procalcitonin level, daily fluid intake/output and balance, and therapeutic interventions (surgery, antibiotics, steroids, diuretics, renal replacement therapy, and so forth) were recorded daily.

All patients were followed for 28 days after enrollment and assessed for 28-day all-cause mortality.

### Determination of pathophysiological diagnostic differential of respiratory insufficiency

At least three experts (intensive care, respiratology, and cardiology) retrospectively determined the pathophysiological mechanism of respiratory insufficiency: ALI/ARDS, increased pulmonary vascular permeability without or with increased pulmonary vascular hydrostatic pressure; cardiogenic edema, increased pulmonary capillary hydrostatic pressure without increased vascular permeability; and pleural effusion with atelectasis, no evidence of lung edema secondary to increased hydrostatic pressure or vascular permeability, as previously reported [7,44]. For this purpose, the experts particularly considered the patients' medical history, clinical presentation and the course, chest computed tomography and radiography findings, echocardiography findings, and serum BNP or *N*-terminal pro-BNP and procalcitonin concentrations, and systemic inflammatory status. The pulmonary capillary wedge pressure was not routinely obtained in this study and any pressures measured depended on the attending physicians' discretion and was only obtained for selected patients. The physicians also considered in particular the time course of all the preceding findings, including daily fluid intake/output and balance, hemodynamic parameters obtained from the thermodilution method, the requirement for systemic management and respiratory therapy, and the clinical responses to these treatments. In this study, EVLWI ≥ 10 ml/kg was used for definition of pulmonary edema, and patients with EVLWI ≥ 10 ml/kg were discriminated between permeability and hydrostatic pulmonary edema. The experts who provided the final diagnosis were completely blinded to the PVPI, but not to hemodynamic parameters such as cardiac index, stroke volume index, ITBV and EVLWI.

### Statistical analysis

Data are presented as mean ± standard error or as median (interquartile range) depending on the distribution normality of the variables. Spearman's rank correlation was used for determining the correlation between two variables, and Mann-Whitney's *U *test was used for assessing the differences between two groups. For multiple-group comparison, analysis of variance on ranks with a Tukey honestly significant difference test was used. Receiver operating characteristic curves were generated for PVPI and ITBV by varying the discriminating threshold of each parameter. The area under the receiver operating characteristic curve for each parameter was calculated and compared using a Hanley-McNeil test. *P *< 0.05 was considered significant. All statistical analyses were performed using SPSS 19.0 for Windows (SPSS, Chicago, IL, USA).

## Results

### Patient characteristics

The most frequent condition leading to exclusion of patients was respiratory insufficiency (P/F ratio ≤ 300 mmHg and slight bilateral infiltration) secondary to sepsis suggesting ALI but not accompanied by EVLWI ≥ 10 ml/kg - the predefined value for pulmonary edema, due to hypovolemia. Consensus on inclusion of such patients was not obtained from all attending experts.

For this analysis, 266 patients were included and divided into the following three categories on the basis of the pathophysiological diagnostic differentiation of the respiratory insufficiency: ALI/ARDS, cardiogenic edema (including fluid overload), and pleural effusion with atelectasis. ALI/ARDS complicated by increased hydrostatic pressure was judged as ALI/ARDS.

Table 1 presents the patient characteristics at the time of enrollment. No patient with body mass index ≥ 30 was included. The incidence of sepsis as a baseline condition was higher in the ALI/ARDS patients than in cardiogenic edema and pleural effusion with atelectasis patients. The period of ventilator-free days within 28 days was significantly longer in patients with cardiogenic edema. Mortality was assessed by 28-day all-cause mortality and there was no significant difference between the three groups. On the day of enrollment, ALI/ARDS patients had higher Acute Physiology and Chronic Health Evaluation II and Sequential Organ Failure Assessment scores than patients with cardiogenic edema, and had higher Lung Injury Scale scores than patients with pleural effusion with atelectasis. ITBV was highest in the cardiogenic edema patients.

**Table 1:** Patient characteristics

Variable	All (*n *= 266)	ALI/ARDS (*n *= 207)	Cardiogenic edema (*n *= 26)	Pleural effusion with atelectasis (*n *= 33)
Age (years)	67.3 ± 16.2	66.7 ± 16.8	70.0 ± 12.5	69.4 ± 14.3
Male	175 (65.3%)	134 (64.7%)	14 (53.8%)	27 (77.1%)
Height (cm)	159.8 ± 10.0	159.4 ± 10.0	157.8 ± 10.4	163.4 ± 9.3
Body weight (kg)	55.2 ± 10.6	54.8 ± 10.6	52.8 ± 11.1	59.2 ± 9.8
BSA (m^2^)	1.56 ± 0.20	1.55 ± 0.20	1.52 ± 0.21	1.64 ± 0.18
Body mass index	21.3 ± 1.8	21.3 ± 1.8	20.9 ± 1.9	22.0 ± 1.5
Heart rate (beats/minute)	102 ± 25	103 ± 22	95 ± 26	103 ± 37
MAP (mmHg)	77 ± 18	76 ± 17	80 ± 18	79 ± 20
Vasopressor	180 (70.7%)	148 (71.5%)*	10 (38.5%)^##^	22 (66.7%)
Sepsis	144 (54.1%)	128 (61.8%)*^,#^	4 (15.4%)	12 (36.3%)
APACHE II score (points)	22.7 ± 8.0	23.4 ± 8.1**	19.7 ± 6.5	20.7 ± 7.7
SOFA score (points)	10.4 ± 3.7	10.7 ± 3.6*	8.1 ± 3.9	10.3 ± 3.5
Respiratory	1.2 ± 1.2	1.2 ± 1.3	0.77 ± 1.1	1.5 ± 0.9
Coagulation	0.9 ± 1.2	0.9 ± 1.1	1.0 ± 1.5	0.9 ± 1.1
Liver	2.3 ± 1.7	2.5 ± 1.6*	1.3 ± 1.6^##^	2.4 ± 1.7
Cardiovascular	0.7 ± 1.0	0.7 ± 1.0	0.4 ± 0.8	0.8 ± 1.0
Central nervous system	3.0 ± 0.8	3.1 ± 0.8*	2.8 ± 0.7	2.7 ± 0.7
Renal	2.3 ± 1.4	2.4 ± 1.4	2.0 ± 1.3	2.0 ± 1.5
PEEP (cmH_2_O)	8.6 ± 4.5	8.7 ± 4.7	7.9 ± 4.2	8.9 ± 3.5
Plateau pressure (cmH_2_O)	22.1 ± 5.7	22.3 ± 5.6	20.6 ± 6.9	21.5 ± 5.2
PaO_2_/FIO_2 _ratio (mmHg)	155.3 ± 70.7	150.5 ± 70.9	166.7 ± 69.8	176.2 ± 67.5
Lung Injury Score (points)	2.3 ± 0.6	2.3 ± 0.6^#^	2.3 ± 0.6	1.8 ± 0.7
Ejection fraction (%)	55.1 ± 13.1*	56.4 ± 12.1*	46.8 ± 13.0	55.0 ± 17.0
Stroke volume variation (%)	15 ± 7	16 ± 7*	11 ± 6^##^	16 ± 7
Cardiac index (l/m^2^/minute)	3.4 ± 1.2	3.5 ± 1.3	3.0 ± 1.0	3.4 ± 1.2
ITBV (ml/m^2^)	1040 ± 303	1021 ± 257*	1312 ± 527^##^	948 ± 215
EVLWI (ml/kg)	16.8 ± 7.1	18.5 ± 6.8*^,#^	14.4 ± 4.0^#^	8.3 ± 2.1
PVPI	2.9 ± 1.4	3.2 ± 1.4*^,#^	2.0 ± 0.8	1.6 ± 0.5
Maximal EVLWI (ml/kg)	19.2 ± 8.5	21.2 ± 8.1*^,#^	16.4 ± 7.5^#^	9.4 ± 2.1
Maximal PVPI	3.4 ± 1.6	3.7 ± 1.6*^,#^	2.2 ± 1.2	1.9 ± 0.6
VFD within 28 days (days)	11.3 ± 10.3	9.8 ± 9.9*	20.3 ± 8.0^##^	13.1 ± 10.7
28-day mortality	99 (37.2%)	84 (40.6%)	7 (26.9%)	(24.2%)

Data presented as mean ± standard deviation or *n *(%). Data on the day of enrollment were presented, except for maximal extravascular lung water index (EVLWI), and maximal pulmonary vascular permeability index (PVPI) during the 3-day study period. ALI/ARDS, acute lung injury/acute respiratory distress syndrome; APACHE, Acute Physiology and Chronic Health Evaluation; BSA, body surface area; ITBV, intrathoracic blood volume index; MAP, mean arterial pressure; PEEP, positive end-expiratory pressure; SOFA, Sequential Organ Failure Assessment; VFD, ventilator-free days. *P < 0.01 vs. cardiogenic edema, **P < 0.05 vs. cardiogenic edema, ^#^*P *< 0.01 vs. pleural effusion with atelectasis, ^##^*P *< 0.05 vs. pleural effusion with atelectasis.

Table 2 shows the underlying condition and mechanism in patients with ALI/ARDS, with 128 of 207 cases caused by sepsis. The most frequent site of infection was the respiratory tract, and in 125 patients the injury was direct.

**Table 2:** Underlying conditions and mechanism for patients with acute lung injury/acute respiratory distress syndrome

Sepsis (*n*)	128
Respiratory	83
Abdomen	26
Urinary tract	5
Soft tissue	3
Others	11
Non-infectious condition (*n*)	79
Trauma	11
Surgery	8
Burn	7
Respiratory diseases	10
Gastrointestinal diseases	5
Central nervous system	4
Severe acute pancreatitis	3
Vascular diseases	2
Others	29
Direct or indirect lung injury	
Direct injury	125
Indirect injury	82

### Comparison of extravascular lung water index and pulmonary vascular permeability index

**Figure 2:**
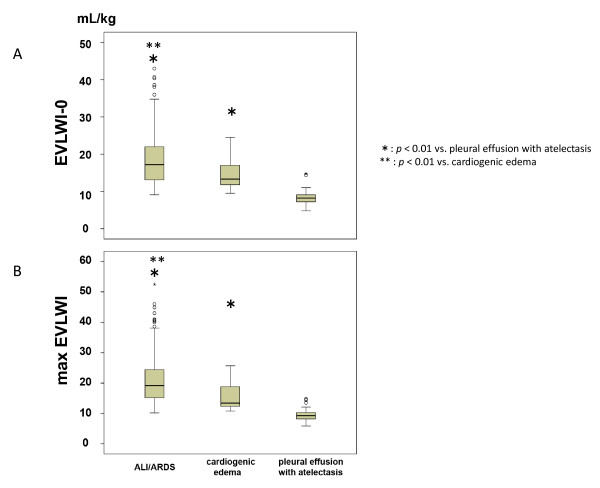
**Comparison of extravascular lung water indexed to predicted body weight**. Comparison of extravascular lung water indexed to predicted body weight of patients with acute lung injury (ALI)/acute respiratory distress syndrome (ARDS), cardiogenic edema, and pleural effusion with atelectasis on the day of enrollment and the maximal value during the study period. **(A) **Extravascular lung water indexed to predicted body weight (EVLWI) on the day of enrollment was significantly higher in ALI/ARDS patients than in pleural effusion with atelectasis patients and cardiogenic edema patients. EVLWI was also higher in cardiogenic edema patients than in pleural effusion with atelectasis patients. **(B) **Differences were found when the maximal EVLWI value was compared between day 0 and day 2. Data presented as median (interquartile range). *P < 0.01 vs. pleural effusion with atelectasis. **P < 0.01 vs. cardiogenic edema. EVLWI-0, extravascular lung water index on day of enrollment; maxEVLWI, maximal value of extravascular lung water index from days 0 to 2.

**Figure 3:**
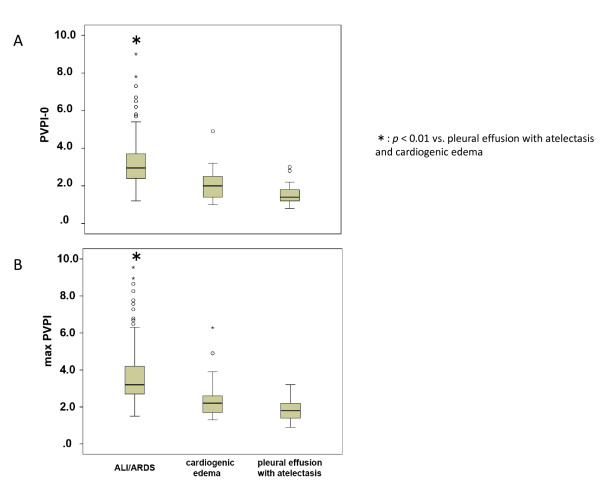
**Comparison of pulmonary vascular permeability index**. Comparison of pulmonary vascular permeability index (PVPI) of patients with acute lung injury (ALI)/acute respiratory distress syndrome (ARDS), cardiogenic edema, and pleural effusion with atelectasis on the day of enrollment and the maximal value during the study period. **(A) **PVPI was higher in ALI/ARDS patients than in cardiogenic edema and pleural effusion with atelectasis patients. There was no difference in the index between cardiogenic edema and pleural effusion with atelectasis patients. **(B) **Differences were found when the maximal index value was compared between day 0 and day 2. Data presented as median (interquartile range). *P < 0.01 vs. pleural effusion with atelectasis and cardiogenic edema. PVPI-0, pulmonary vascular permeability index on the day of enrollment; maxPVPI, maximal value of pulmonary vascular permeability index from days 0 to 2.

The EVLWI on the day of enrollment was significantly higher in ALI/ARDS patients than in patients with pleural effusion with atelectasis (18.5 ± 6.8 vs. 8.3 ± 2.1; *P *< 0.01) or cardiogenic edema (14.4 ± 4.0; *P *< 0.01) (Figure 2). The PVPI on the day of enrollment was higher in the ALI/ARDS patients than in cardiogenic edema or pleural effusion with atelectasis patients (3.2 ± 1.4, 2.0 ± 0.8, and 1.6 ± 0.5, respectively). Although the EVLWI was higher in the cardiogenic edema than in pleural effusion with atelectasis patient (Figure 2), there was no significant difference in PVPI between those groups (Figure 3).

These differences were also noted when the maximal values of EVLWI and PVPI recorded during the study period were compared among the three groups (Figures 2 and 3).

### Relationship among EVLWI, PVPI, and intrathoracic blood volume

For this analysis, cardiogenic edema and pleural effusion with atelectasis patients were considered non-ALI/ARDS patients because increased pulmonary vascular permeability is not the pathogenetic mechanism of these conditions and was not elevated compared with that in ALI/ARDS patients.

**Figure 4:**
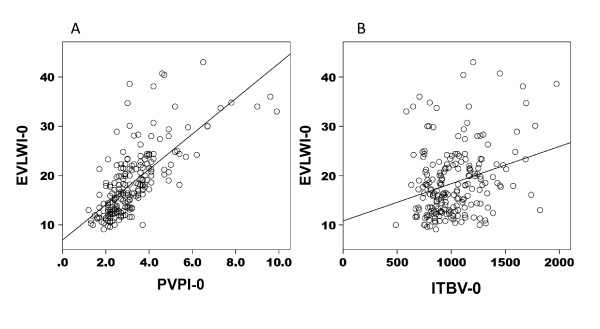
**Extravascular lung water index and pulmonary vascular permeability index/intrathoracic blood volume correlation in ALI/ARDS patients**. Correlation between extravascular lung water index (EVLWI) and pulmonary vascular permeability index (PVPI) and that between EVLWI and intrathoracic blood volume (ITBV) in patients with acute lung injury/acute respiratory distress syndrome. There was a strong correlation between EVLWI and PVPI (*r *= 0.729, *P *< 0.01) **(A) **and a weak correlation between EVLWI and ITBV (*r *= 0.236, *p *< 0.01) **(B)**. EVLWI-0, extravascular lung water index on the day of enrollment; PVPI-0, pulmonary vascular permeability index on the day of enrollment; ITBV-0, intrathoracic blood volume on the day of enrollment.

**Figure 5:**
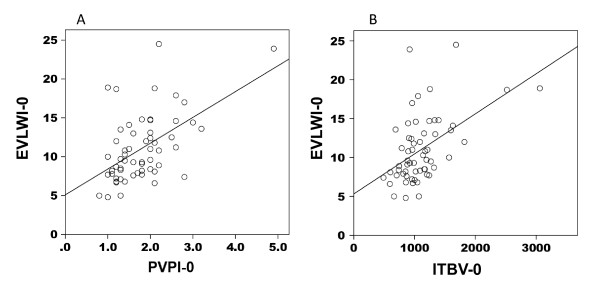
**Extravascular lung water index and pulmonary vascular permeability index/intrathoracic blood volume correlation in non-ALI/ARDS patients**. Correlation between extravascular lung water index (EVLWI) and pulmonary vascular permeability index (PVPI) and that between EVLWI and intrathoracic blood volume (ITBV) in patients with non-acute lung injury (ALI)/acute respiratory distress syndrome (ARDS). For this analysis, cardiogenic edema and pleural effusion with atelectasis patients were combined as non-ALI/ARDS. EVLWI had a moderate correlation with PVPI (*r *= 0.464, *P *< 0.01) **(A) **and with ITBV (*r *= 0.493, *P *< 0.01) **(B)**. EVLWI-0, extravascular lung water index on the day of enrollment; PVPI-0, pulmonary vascular permeability index on the day of enrollment; ITBV-0, intrathoracic blood volume on the day of enrollment.

In the ALI/ARDS patients, a strong correlation between EVLWI and PVPI (*r *= 0.729, *P *< 0.01) and a weak correlation between EVLWI and ITBV (*r *= 0.236, *P *< 0.01) were noted on the day of enrollment (Figure 4). In the non-ALI/ARDS patients, moderate correlations between EVLWI and PVPI (*r = *0.464, *P *< 0.01) and between EVLWI and ITBV (*r *= 0.493, *P *< 0.01) were noted (Figure 5).

Multiple regression analysis was also performed using EVLWI as the dependent variable with PVPI and ITBV as the independent variables. The standardized partial regression coefficients were 0.958 for PVPI and 0.646 for ITBV in ALI/ARDS patients, and were 0.836 and 0.814 in non-ALI/ARDS patients, respectively, suggesting the important contribution of PVPI on the EVLWI in ALI/ARDS.

### Relationship between extravascular lung water index and PaO_2_/FiO_2 _ratio

For this analysis, patients with pleural effusion with atelectasis were excluded because the increased EVLW is not the pathogenetic mechanism of this condition, and EVLWI in these patients was not high as in those patients with ALI/ARDS and cardiogenic edema.

**Figure 6:**
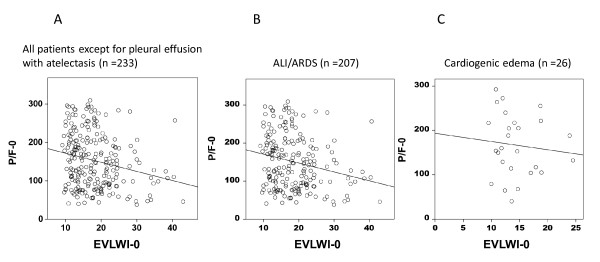
**Correlation between extravascular lung water index and PaO_2_/FiO_2 _ratio**. There was a negative and weak correlation between extravascular lung water index (EVLWI) and PaO_2_/FiO_2 _(P/F) ratio in all patients except for pleural effusion with atelectasis patients (*r *= -0.213, *P *< 0.01) **(A)**. In acute lung injury (ALI)/acute respiratory distress syndrome (ARDS) patients (*n *= 207), a weak correlation between EVLWI and P/F ratio was observed (*r *= -0.215, *P *< 0.01) **(B)**. No correlation between EVLWI and pulmonary vascular permeability index was observed in cardiogenic edema patients (*n *= 26; *r *= -0.176, *P *= 0.39) **(C)**. EVLWI-0, extravascular lung water index on the day of enrollment; P/F-0, PaO_2_/FiO_2 _ratio on the day of enrollment.

The P/F ratio varied widely at all levels of EVLWI in patients with both ALI/ARDS and cardiogenic edema (Figure 6). A negative but weak correlation was noted between EVLWI and the P/F ratio in all patients except for those with pleural effusion with atelectasis (*r *= -0.213, *P *< 0.01) and ALI/ARDS (*r *= -0.215, *P *< 0.01). No correlation was found between EVLWI and PVPI in cardiogenic edema patients (*r *= -0.176, *P *= 0.39).

### Differential diagnosis of ALI/ARDS on the basis of pulmonary vascular permeability

**Figure 7:**
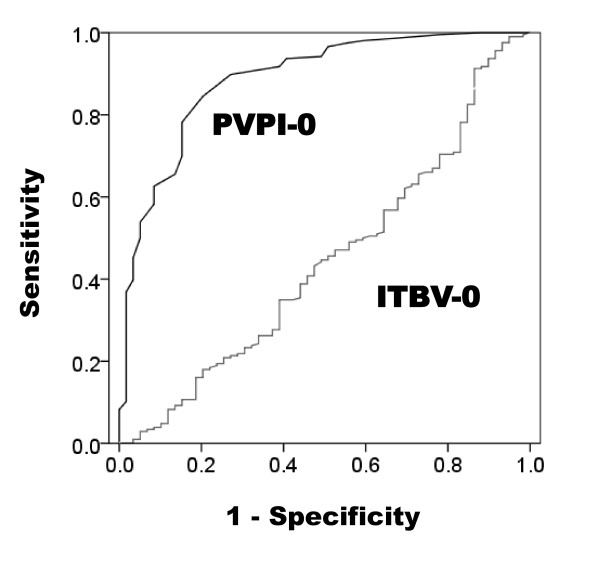
**Receiver operating characteristic curves for pulmonary vascular permeability index and intrathoracic blood volume**. Receiver operating characteristic curves for pulmonary vascular permeability index (PVPI) and intrathoracic blood volume (ITBV) on the day of enrollment for the differential diagnosis of acute lung injury/acute respiratory distress syndrome. The area under the curve for PVPI (0.886; confidence interval, 0.836 to 0.935) was significantly larger than that for ITBI (0.425; confidence interval, 0.359 to 0.529; *P *< 0.01). PVPI-0, pulmonary vascular permeability index on the day of enrollment; ITBV-0, intrathoracic blood volume on the day of enrollment.

Receiver operating characteristic curves were generated using PVPI and ITBV on the day of enrollment to differentiate between ALI/ARDS patients and non-ALI/ARDS patients. The area under the curve for PVPI (0.886; confidence interval, 0.836 to 0.935) was significantly larger than that for ITBV (0.575; confidence interval, 0.471 to 0.651; *P *< 0.01) (Figure 7).

The cutoff value for the definitive quantitative diagnosis of ALI/ARDS needs to be determined considering the high specificity despite the decreased level of sensitivity, as discussed in the next section. The cutoff value of the PVPI to diagnose ALI/ARDS was found to be between 2.85 (sensitivity, 0.54; specificity, 0.95) and 2.6 (sensitivity, 0.64; specificity, 0.90). The cutoff value of the PVPI to diagnose non-ALI/ARDS was between 1.7 (sensitivity, 0.50; specificity, 0.95) and 2.0 (sensitivity, 0.70; specificity, 0.90).

## Discussion

In this prospective multi-institutional observational study, EVLW and pulmonary vascular permeability were assessed by transpulmonary thermodilution in patients requiring mechanical ventilation with P/F ratio ≤ 300 mmHg and bilateral pulmonary infiltration on chest X-ray scan. The results showed that EVLW was greater in patients with ALI/ARDS and cardiogenic edema than in those with pleural effusion with atelectasis; that pulmonary vascular permeability was increased in patients with ALI/ARDS compared with cardiogenic edema and pleural effusion with atelectasis patients; and that EVLW, the crucial pathogenetic factor of pulmonary edema, was weakly correlated with the P/F ratio in patients with ALI/ARDS and cardiogenic edema.

ARDS is associated with a high incidence of morbidity and mortality despite the development of improved management techniques over the past two decades [45]. Difficulties in selecting appropriate patient populations that would benefit from specific treatments occur largely because of the lack of homogeneity in the disease definition. The AECC criteria, which have been exclusively used as the inclusion criteria in clinical trials for ALI/ARDS, were designed to identify patients with ALI/ARDS [9]. These criteria are inclusive, so that the population selected on the basis of these criteria can be very heterogeneous in disease severity and clinical outcomes. This heterogeneity might be the reason why most clinical trials in ARDS have failed to achieve improved mortality. There is no validated biomarker that allows early recognition of increased lung vascular permeability, the hallmark of ALI/ARDS pathogenesis. Although the Berlin definition is expected to have sufficient specificity and acceptable sensitivity, an accurate definition can also help clinicians identify patients who may benefit from precise ventilatory strategies, diagnostic procedures, or drug therapies.

Although pulmonary capillary hydrostatic pressure elevation and pulmonary vascular permeability increase are known to induce pulmonary edema, discrimination of these pathogeneses is important because of the difference in treatments. At present, the differential diagnosis is made on the basis of the assessment of left atrial pressure, which is assumed to be normal in ALI/ARDS [46]. However this hemodynamic definition of ALI/ARDS is controversial, as suggested previously [47]. The pulmonary artery occlusion pressure might not reflect the hydrostatic pressure in the pulmonary micro-vessels [48,49] and cannot be accurately measured [50,51]. Moreover, left ventricular preload might be elevated in ALI/ARDS patients, especially in those who have already received volume resuscitation and/or pre-existing or sepsis-related cardiac dysfunction, as described in the Berlin definition [10]. The pulmonary artery occlusion pressure was recently found to be elevated in 30% of ARDS patients [52]. The definition of ALI/ARDS should thus include the functional features of the pathophysiology of this syndrome; that is, increased pulmonary microvascular permeability [46]. The PVPI has been suggested to be an important parameter in ALI/ARDS pathogenesis [53].

### EVLWI in ALI/ARDS, cardiogenic edema, and pleural effusion with atelectasis

A close agreement between thermodilution EVLW values and gravimetric lung water measurements, which are thought to be the gold standard for the quantitative evaluation of EVLW, has been shown in animal models with lung injury [27,54,55] and in a human autopsy study [25].

In this study, EVLWI ≥ 10 ml/kg was defined as pulmonary edema. Although no definitive quantitative criteria of EVLWI for pulmonary edema were established, we recently reported that the normal EVLWI value is approximately 7.4 ± 3.3 ml/kg from a human autopsy study and this value can distinguish between healthy and pathological lungs [24]. Because EVLWI ≥ 10 ml/kg has been shown to predict progression to acute lung injury, this value was used for defining pulmonary edema in this study [1,29]. Only three patients had EVLWI < 10 ml/kg from the 155 ARDS patients, based on the AECC definition.

Regarding the effect of pleural effusion on EVLW measurements, Blomqvist and colleagues showed that the pleural fluid level did not affect the reliability of the double-indicator dilution technique for measuring EVLW in dogs [56]. This relationship has been also demonstrated using the single indicator thermodilution method in critically ill patients in a previous study [57]. We showed a very close correlation between premortem single-indicator thermodilution measurement of EVLW and postmortem lung weight, regardless of the degree of pleural effusion [25]. In this study, the EVLWI of patients with pleural effusion with atelectasis was less than the defined value of pulmonary edema, despite the fact that patients had P/F ratio ≤ 300 mmHg and bilateral infiltration. This suggests that the EVLWI value might not be significantly influenced by pleural effusion with or without massive atelectasis. If patients have EVLWI < 10 ml/kg but P/F ratio ≤ 300 mmHg and bilateral infiltration, thoracic ultrasound for the evaluation of pleural effusion should be performed.

The EVLWI value was different between ALI/ARDS and cardiogenic edema in this study. Hydrostatic pressure elevation occurred without a significant increase in vascular permeability in patients with cardiogenic edema, whereas both increased vascular permeability and elevated hydrostatic pressure contributed to the development of edema in ALI/ARDS patients. As already suggested, left ventricular preload can be elevated in ALI/ARDS, especially in patients who already have volume resuscitation and/or have pre-existing or sepsis-related cardiac dysfunction [10,52]. In this regard, more than 15% of the ALI/ARDS patients included in this study had a combined mechanism of an elevated left ventricular preload and vascular permeability; this may have contributed to the difference in EVLWI between the two groups. One should also consider that the heterogeneity of the patients' condition may affect the value of the EVLWI, as shown in a recent meta-analysis where the EVLWI in septic patients was higher than in surgical patients (11.0 ml/kg vs. 7.2 ml/kg) [58].

### Determination of the cutoff value for ALI/ARDS diagnosis

Although EVLW has been extensively evaluated as not only a predictor but also a diagnostic and prognostic parameter for ALI/ARDS [59], this value provides only the degree of accumulated EVLW and not the underlying pathophysiological mechanism.

Increased pulmonary vascular permeability can be estimated by the PVPI [53]. A recent retrospective study showed EVLWI values of 22 ± 9 and 16 ± 4 in patients with ARDS and hydrostatic pulmonary edema, respectively [7]. On the contrary, in our study, these values were 18.5 ± 6.8 and 8.3 ± 2.1, respectively. These differences might have influenced the PVPI values obtained in both studies (4.7 ± 1.8 vs. 3.2 ± 1.4 in ALI/ARDS and 2.1 ± 0.5 vs. 2.0 ± 0.8 in cardiogenic edema). Although the previous study did not show the Acute Physiology and Chronic Health Evaluation II score and mortality, the difference in disease severity and EVLWI values for inclusion or definition of pulmonary edema might have influenced the determined cutoff value for ALI/ARDS. However, the proposed cut-off value of PVPI ≥ 3 for the diagnosis of ALI/ARDS was almost the same as the present study, suggesting that this multicenter study confirmed the proposed value.

The cutoff value for ALI/ARDS should be determined by considering the balance between sensitivity and specificity. Clinical trials focusing on specific therapy for ALI/ARDS should select cutoff values with higher specificity, whereas those focusing on the early recognition of this condition and therapeutic intervention should select cutoff values with high sensitivity. Because the procedures for measuring EVLW and pulmonary vascular permeability are not non-invasive, the validity of the definite diagnosis of ALI/ARDS made on the basis of PVPI values that have high specificity should be considered.

### Limitations

The mechanism underlying respiratory insufficiency - that is, permeability pulmonary edema, cardiogenic edema, or pleural effusion with atelectasis, which may be exclusive or overlapped - was defined by expert consensus, and subjectivity therefore cannot be completely ruled out. Nonetheless, only those patients who were considered eligible by all the experts were included in this study and 35 patients were excluded from the analysis. There may have been some statistical bias in this regard and the patient population may not represent the general population of mechanically ventilated patients with hypoxemic respiratory failure and radiographic infiltrates. Fourteen of the 35 excluded patients were judged to have respiratory failure secondary to sepsis-induced increased pulmonary vascular permeability but had to be excluded because they presented EVLWI < 10 ml/kg. This exclusion may bias the study.

Although the study sample size was not small, the number of patients with cardiogenic pulmonary edema was fewer than those with ALI/ARDS. Most patients with cardiogenic pulmonary edema are managed using non-invasive positive pressure ventilation without tracheal intubation; hence, these patients could not be included in this study. There may have been some statistical bias in this regard.

Pulmonary inflammation (that is, pneumonia) needs to be considered because it might influence the thermodilution technique findings. Inflamed cells and purulent matter, including microabscesses, may increase lung weight despite increased EVLW. Further evaluation may be required to assess ALI/ARDS secondary to direct injury from pneumonia.

EVLWI ≥ 10 ml/kg was used for defining pulmonary edema in this study. Although no definitive quantitative criteria for pulmonary edema were established, this value was selected on the basis of the value found in our recent human autopsy study and those used for defining pulmonary edema in previously reported studies. Lowering the EVLWI value for pulmonary edema may have led to the inclusion of less severe pulmonary edema cases and might have influenced the diagnostic cutoff value and its accuracy. However, most of the patients who had pleural effusion with atelectasis showed EVLWI < 10 ml/kg, suggesting that the value was not too low.

## Conclusion

This study showed that EVLW was greater in patients with ALI/ARDS and cardiogenic edema than patients with pleural effusion with atelectasis; that pulmonary vascular permeability was increased in patients with ALI/ARDS compared with cardiogenic edema and pleural effusion with atelectasis patients; and that the cutoff value of PVPI for the quantitative diagnosis of ALI/ARDS was between 2.6 and 2.85, with a specificity of 0.9 to 0.95, and that PVPI < 1.7 ruled out an ALI/ARDS diagnosis (specificity, 0.95).

## Key messages

• EVLW was greater in patients with ALI/ARDS and cardiogenic edema than patients with pleural effusion with atelectasis.

• Pulmonary vascular permeability was increased in patients with ALI/ARDS compared with cardiogenic edema and pleural effusion with atelectasis patients.

• The cutoff value of the PVPI for the quantitative diagnosis of ALI/ARDS was between 2.6 and 2.85, with a specificity of 0.9 to 0.95, and PVPI < 1.7 ruled out an ALI/ARDS diagnosis (specificity, 0.95).

## Abbreviations

AECC: American-European Consensus Conference; ALI: acute lung injury; ARDS: acute respiratory distress syndrome; BNP: B-type natriuretic peptide; CO: cardiac output; EVLW: extravascular lung water; EVLWI: extravascular lung water index; FiO_2_: fraction of inspired oxygen; GEDV: global end-diastolic volume; ITBV: intrathoracic blood volume; PaO_2_: partial pressure of arterial oxygen; P/F ratio: PaO_2_/FiO_2 _ratio; PVPI: pulmonary vascular permeability index.

## Competing interests

YT is a member of the medical advisory board of Pulsion Medical Systems. The remaining authors declare that they have no competing interests.

## Authors' contributions

All authors conceived and designed the study, wrote the study protocol and contributed to clinical data acquisition. The statistical analyses and the first draft of manuscript were performed by SK. All authors amended and commented on the manuscript and approved the final version.
